# Artificial Intelligence in Cardiac Surgery: Transforming Outcomes and Shaping the Future

**DOI:** 10.3390/clinpract15010017

**Published:** 2025-01-14

**Authors:** Vasileios Leivaditis, Eleftherios Beltsios, Athanasios Papatriantafyllou, Konstantinos Grapatsas, Francesk Mulita, Nikolaos Kontodimopoulos, Nikolaos G. Baikoussis, Levan Tchabashvili, Konstantinos Tasios, Ioannis Maroulis, Manfred Dahm, Efstratios Koletsis

**Affiliations:** 1 Department of Cardiothoracic and Vascular Surgery, WestpfalzKlinikum, 67655 Kaiserslautern, Germany; vleivaditis@westpfalz-klinikum.de (V.L.); apapatriantafy@westpfalz-klinikum.de (A.P.); mdahm@westpfalz-klinikum.de (M.D.); 2 Department of Anesthesiology and Intensive Care, Hannover Medical School, 30625 Hannover, Germany; beltsios.eleftherios@mh-hannover.de; 3 Department of Thoracic Surgery and Thoracic Endoscopy, Ruhrlandklinik, West German Lung Center, University Hospital Essen, University Duisburg-Essen, 45141 Essen, Germany; grapatsaskostas@gmail.com; 4 Department of General Surgery, General University Hospital of Patras, 26504 Patras, Greece; levatcha@pgnp.gr (L.T.); ceid4551@ac.upatras.gr (K.T.); 5 Department of Economics and Sustainable Development, Harokopio University, 17778 Athens, Greece; nkontodi@otenet.gr; 6 Department of Cardiac Surgery, Ippokrateio General Hospital of Athens, 11527 Athens, Greece; nikolaos.baikoussis@gmail.com; 7 Department of Cardiothoracic Surgery, General University Hospital of Patras, 26504 Patras, Greece; ekoletsis@med.upatras.gr

**Keywords:** artificial intelligence, cardiac surgery, machine learning, robotic-assisted surgery, risk stratification, augmented cognition, postoperative management

## Abstract

**Background:** Artificial intelligence (AI) has emerged as a transformative technology in healthcare, with its integration into cardiac surgery offering significant advancements in precision, efficiency, and patient outcomes. However, a comprehensive understanding of AI’s applications, benefits, challenges, and future directions in cardiac surgery is needed to inform its safe and effective implementation. **Methods:** A systematic review was conducted following PRISMA guidelines. Literature searches were performed in PubMed, Scopus, Cochrane Library, Google Scholar, and Web of Science, covering publications from January 2000 to November 2024. Studies focusing on AI applications in cardiac surgery, including risk stratification, surgical planning, intraoperative guidance, and postoperative management, were included. Data extraction and quality assessment were conducted using standardized tools, and findings were synthesized narratively. **Results:** A total of 121 studies were included in this review. AI demonstrated superior predictive capabilities in risk stratification, with machine learning models outperforming traditional scoring systems in mortality and complication prediction. Robotic-assisted systems enhanced surgical precision and minimized trauma, while computer vision and augmented cognition improved intraoperative guidance. Postoperative AI applications showed potential in predicting complications, supporting patient monitoring, and reducing healthcare costs. However, challenges such as data quality, validation, ethical considerations, and integration into clinical workflows remain significant barriers to widespread adoption. **Conclusions:** AI has the potential to revolutionize cardiac surgery by enhancing decision making, surgical accuracy, and patient outcomes. Addressing limitations related to data quality, bias, validation, and regulatory frameworks is essential for its safe and effective implementation. Future research should focus on interdisciplinary collaboration, robust testing, and the development of ethical and transparent AI systems to ensure equitable and sustainable advancements in cardiac surgery.

## 1. Introduction

Artificial intelligence (AI) is revolutionizing healthcare by enabling machines to perform cognitive tasks such as problem solving, decision making, and language processing, traditionally reliant on human intelligence [1]. In healthcare, AI systems leverage large datasets to identify patterns, make predictions, and support clinical decisions, improving diagnostic accuracy, treatment planning, and patient outcomes. From automated image analysis to personalized medicine, AI is being integrated across diverse medical fields, significantly enhancing efficiency and quality of care [2,3].

The role of AI is particularly prominent in surgery, where it addresses the complexities of decision making by analyzing patient-specific factors such as anatomy, disease progression, risk profiles, and costs. AI assists surgeons by providing predictive models, real-time insights, and enhanced imaging techniques that minimize human error and improve surgical precision. Technologies such as machine learning (ML), computer vision, and robotic systems are transforming surgical planning, intraoperative guidance, and postoperative management, marking a paradigm shift in surgical care delivery [4,5].

In the field of cardiovascular medicine, AI has demonstrated remarkable potential, particularly in the management of cardiac diseases and surgical interventions. Cardiovascular diseases (CVDs) remain the leading cause of mortality worldwide, demonstrating the need for innovative approaches to diagnosis and treatment. AI has been applied in diverse aspects of cardiac care, from risk stratification and imaging analysis to predicting surgical outcomes. For instance, AI-driven algorithms outperform traditional statistical models in predicting complications such as acute kidney injury and mortality following cardiac surgery [6,7]. Furthermore, AI enhances preoperative planning through automated echocardiography and cardiac CT analysis, enabling more precise assessments of anatomical structures and procedural strategies [8].

This review aims to provide a comprehensive analysis of AI’s applications in cardiac surgery, focusing on its current integration into clinical practice and its potential to enhance surgical precision, efficiency, and patient outcomes. The scope includes a discussion of AI’s role in preoperative diagnostics, intraoperative assistance, and postoperative management, as well as its impact on healthcare systems globally. By synthesizing existing evidence, this review seeks to highlight the transformative potential of AI in cardiac surgery while addressing the challenges and future directions for its implementation.

## 2. Materials and Methods

This study was intended as a comprehensive narrative review of artificial intelligence applications in cardiac surgery. However, systematic review principles and guidelines were followed and applied to enhance its methodological rigor. The PRISMA framework was adapted to provide a transparent and structured approach for identifying, screening, and including relevant studies.

A systematic literature review was conducted to evaluate the applications and impact of artificial intelligence (AI) in cardiac surgery. Searches were performed across five major databases: PubMed, Scopus, Cochrane Library, Google Scholar, and Web of Science. The search period spanned publications from January 2000 to November 2024 to capture the development and recent advancements in the field. The search strategy combined keywords and Boolean operators tailored to the topic, including “Artificial intelligence” AND “cardiac surgery”, “Machine learning” AND “cardiovascular diseases”, “AI-assisted surgery” OR “robotic surgery”, “Real-time decision support” AND “cardiac care”, “Deep learning” AND “risk stratification”, “Cardiac imaging” OR “AI-guided echocardiography”, and “Ethical considerations” AND “AI in surgery”. Additional filters for language (English only) and study type (clinical trials, reviews, observational studies, and meta-analyses) were applied. The reference lists of included articles were screened for additional relevant studies.

Inclusion and exclusion criteria were defined to ensure the relevance and quality of the studies reviewed.

Inclusion Criteria:

- Studies examining AI applications in cardiac surgery or related fields.
- Peer-reviewed publications.
- Research focusing on AI technologies such as machine learning, deep learning, computer vision, and robotics.
- Articles discussing preoperative planning, intraoperative assistance, or postoperative management in cardiac surgery.
- Studies addressing ethical, practical, or regulatory considerations in AI use for cardiac surgery.

Exclusion Criteria:

- Non-peer-reviewed articles, conference abstracts, and editorials.
- Studies focusing solely on non-cardiac surgical specialties.
- Articles without quantitative or qualitative data supporting AI application.
- Duplicate studies across databases.
- Data extraction and analysis

The inclusion criteria were designed to ensure that the selected studies provided robust and relevant evidence on the role of AI in cardiac surgery. By focusing on peer-reviewed articles, we ensured that only high-quality research with established academic rigor was included. The requirement for studies to examine AI applications specifically in cardiac surgery ensured a focused review relevant to the field, while including a variety of AI technologies allowed us to capture the breadth of innovations in this area. Studies addressing ethical, practical, or regulatory challenges were included to provide a balanced perspective on the integration of AI.

The exclusion criteria were equally stringent to maintain the review’s credibility. The non-peer-reviewed literature and articles without quantitative or qualitative data were excluded to avoid anecdotal or unsupported claims. Similarly, studies focusing on non-cardiac surgical specialties or duplications across databases were removed to maintain relevance and eliminate redundancy. This rigorous process ensured that the studies included in the review were both relevant and scientifically sound. All included studies underwent a structured quality assessment using tools such as the Newcastle–Ottawa Scale (NOS) for observational studies and the Cochrane Risk of Bias Tool for randomized controlled trials. Studies were rated as low, moderate, or high risk of bias, with only low to moderate risk studies included in the synthesis.

**Figure 1:**
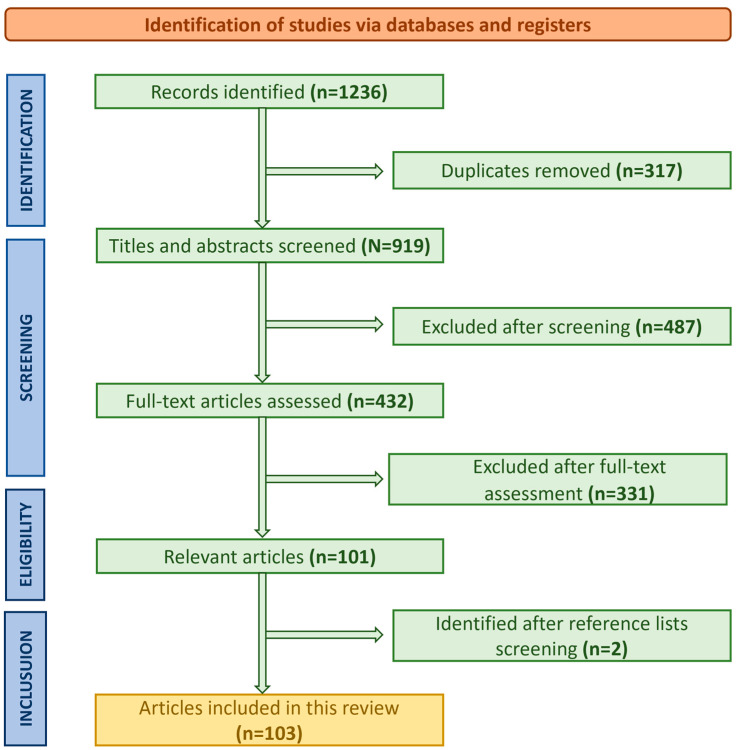
PRISMA flow diagram outlining the identification, screening, eligibility, and inclusion process of studies for this systematic review on artificial intelligence in cardiac surgery. A total of 1236 records were identified, with 103 studies meeting the inclusion criteria following rigorous screening and eligibility assessments.

After removing duplicates, a total of 1236 studies were identified. Following title and abstract screening, 432 studies met the inclusion criteria for full-text review. Reference lists were manually screened for additional studies. Ultimately, 103 studies were included in the analysis, comprising clinical trials, systematic reviews, and observational studies. The study selection process, including identification, screening, and inclusion of relevant articles, is summarized in the PRISMA flow diagram (Figure 1). Standardized forms were used to extract data on study design, sample size, AI methodology, outcomes, and other relevant factors. Data were organized in Microsoft Excel to ensure consistency and facilitate synthesis. To provide a comprehensive overview of the role of artificial intelligence in cardiac surgery, we systematically identified and reviewed relevant studies, summarized in Table 1, highlighting their key outcomes and contributions to the field.

Searches were conducted using database-specific tools such as PubMed’s Advanced Search Builder, Scopus Query Builder, and Cochrane Library’s search tool. The screening process was managed using Rayyan QCRI, a systematic review platform, to organize studies and facilitate collaboration. Data extraction was performed using standardized forms developed in Microsoft Excel, capturing information such as study design, sample size, AI methodology, and outcomes.

To ensure rigor and reliability, the quality of the included studies was assessed using the PRISMA (Preferred Reporting Items for Systematic Reviews and Meta-Analyses) guidelines, which were followed for study identification and selection. Although this study was not initially designed as a systematic review, it is a narrative review with a systematic search strategy and adherence to PRISMA guidelines ensured a systematic and transparent approach to study selection and reporting. The PRISMA flow diagram reflects the rigor of the methodology and the comprehensiveness of the review process.

The review encompasses studies evaluating AI’s role in preoperative planning, intraoperative assistance, and postoperative management. Specific themes analyzed include the following:

- Risk stratification and outcome prediction using machine learning.
- AI-guided imaging and diagnostic technologies.
- Robotic-assisted and computer vision-based surgical systems.
- Ethical and regulatory challenges in AI adoption.
- Future trends and research opportunities in AI for cardiac surgery

## 3. Discussion

### 3.1. Benefits and Novel Contributions of Artificial Intelligence in Cardiac Surgery

#### 3.1.1. Risk Stratification

Cardiac surgery carries significant risks, making precise risk stratification critical for decision making. Traditional tools such as EuroSCORE II and STS scores are widely used but cannot replace clinical judgment and multidisciplinary discussions [9,10,11]. Machine learning (ML) offers a more sophisticated alternative, with algorithms surpassing traditional statistical methods in predicting in-hospital mortality and postoperative complications [9,12,13]. For example, ML models have shown superior accuracy in identifying prognostic variables and generating diagnostic functions, though issues such as data bias, calibration, and external validation remain challenges [10,13,14].

ML-based approaches have proven particularly valuable in specific contexts. For instance, a gradient boosting algorithm developed by Lee et al. provided the highest predictive accuracy for acute kidney injury (AKI), with an Internet-based estimator offering real-time risk assessments [12]. Similarly, Allyn et al. demonstrated that ML models significantly outperformed EuroSCORE II and logistic regression in predicting mortality after elective cardiac surgery, highlighting their potential for routine use in clinical settings [9]. However, findings by Penny-Dimri et al. suggest that ML methods do not consistently outperform traditional models due to data limitations and slow adoption [15,44].

In heart valve surgery, ML tools such as random forests and support vector machines have improved risk classification accuracy. However, their incremental value over surgeons’ intuition remains debated, and further prospective validation is needed in diverse clinical settings [16,17]. For transcatheter aortic valve implantation (TAVI), ML has demonstrated remarkable accuracy in predicting mortality, achieving AUC values of 0.94–0.97, far surpassing traditional scores such as STS/ACC TAVR. Nevertheless, these models are less effective at predicting other complications, such as stroke and vascular issues [17,18].

AI has also shown promise in early detection of complications. A model developed by Kalisnik et al. achieved high sensitivity and specificity for identifying AKI within 12 h post-surgery, representing a significant advancement in postoperative care [19]. Similarly, AI-based tools for detecting delirium in cardiac surgery patients have proven effective in identifying underrecognized cases, emphasizing their utility in enhancing perioperative monitoring [20].

In heart transplantation, AI is being explored to optimize organ use and predict post-transplant outcomes. Studies reveal that ML algorithms outperform traditional methods in predicting mortality and complications, though challenges with data interpretation and generalizability persist. For example, self-explaining neural networks achieved similar predictive accuracy as deep learning models for 1-year mortality, highlighting the potential of transparent, interpretable AI tools in clinical decision support [21,22,45].

In summary, ML has shown superior accuracy in mortality prediction and risk stratification compared with traditional models, with applications extending to specific challenges in cardiac surgery, such as AKI detection and transplantation outcomes. However, continued refinement and validation across diverse datasets are essential for broader clinical adoption.

#### 3.1.2. Enhanced Surgical Planning

AI has transformed surgical planning by enabling the creation of virtual 3D replicas of a patient’s anatomy, providing a detailed understanding of their condition and assisting in selecting optimal surgical approaches [23,24,46]. These virtual models allow surgeons to simulate procedures, improving accuracy and outcomes by addressing potential challenges before the actual operation. For example, in cases of aortic dissection, AI-driven mathematical models using patient-specific data can predict the risk of rupture and recommend preventive surgical strategies [25,26,47,48].

Traditional surgical planning often relies on medical imaging interpreted during multidisciplinary team (MDT) meetings. However, cognitive biases and variations in interpretation may affect decision making, and plans are typically confined to the operating team without broader input [27,49,50]. The integration of AI minimizes these limitations by providing objective and reproducible analyses, enhancing decision-making processes.

In cardiac surgery, precise planning is critical due to the high-risk nature of procedures and the potential for even minor errors to have severe consequences [28,51]. AI enhances this process by integrating imaging and biophysical data with surgical databases, surpassing traditional models such as logistic regression in heart valve research [17]. As AI evolves, it holds the potential to enable real-time biophysical integration across pre-, intra-, and postoperative phases, reducing costs, minimizing errors, and improving patient outcomes.

#### 3.1.3. Improved Surgical Accuracy

AI enhances surgical accuracy by reducing technical errors and improving decision making through data-driven simulations. Current AI tools achieve reliability rates of 95–98%, compared with the 85% accuracy typical of traditional methods, by leveraging statistical probabilities and virtual simulations to optimize surgical outcomes. For example, the Da Vinci Surgical System, though not fully autonomous, allows surgeons to perform minimally invasive procedures with greater precision, reducing complications by 8.2%, hospital stays by 10%, and blood transfusion needs by 79% compared with traditional methods [52,53,54,55]. However, the transition to fully autonomous AI-guided surgery raises ethical and practical questions about its desirability [56,57].

AI can further enhance surgical decision making through multi-modal data analysis. Systems such as CORRIBA, which analyzes coronary angiograms to recommend revascularization strategies, have shown promise in matching expert panel decisions and are expected to offer superior evidence-based advice in the future [58,59]. Additionally, robotic platforms such as NAVIO and MAKO use haptic feedback to assist surgeons in maintaining precise resection or prosthesis placement, preventing errors by warning against deviations from preoperative plans [60,61].

AI’s ability to process real-time data is particularly valuable for managing complex surgeries. By focusing on reducing technical errors, which occur in up to 39% of all surgeries and are preventable in 18%, AI improves safety, reduces morbidity, and enhances cost-effectiveness [62,63]. Structural heart disease (SHD) highlights the need for advanced tools that go beyond traditional imaging, emphasizing planning and simulation. The integration of AI with 3D printing and computational modeling has shortened the learning curve for transcatheter interventions and improved understanding of cardiac pathophysiology and device interactions, advancing patient-centered care [18,29].

In specific procedures such as coronary artery bypass grafting, AI tools such as intraoperative transit-time flow (TTF) measurements show potential for predicting graft failure. For example, studies have identified parameters such as mean flow and pulsatility index as key predictors of patency, though retrospective biases and limited applicability to broader practice warrant further research [64].

In summary, AI-driven tools and robotic platforms enhance surgical accuracy by enabling precise execution, minimizing errors, and advancing procedural planning. However, ongoing research and validation are critical for expanding their clinical adoption.

#### 3.1.4. Real-Time Monitoring and Decision Support

Advancements in technology have transformed surgical tasks in modern operating rooms (ORs), integrating AI systems to optimize workflows and support surgical teams. By analyzing diverse data—including patient risk factors, anatomy, medical history, and costs—AI assists in making more informed predictions about surgical outcomes, thereby enhancing decision making [30,31].

The implementation of AI in real-time monitoring and decision support remains a complex process requiring a patient-centered approach. Development must prioritize improving patient outcomes and remain adaptable to evidence-based refinements as unintended consequences and uncertainties in efficacy are common during adoption [65,66]. AI systems have significant potential to deliver efficient, cost-effective care while continuously monitoring patients throughout the course of complex diseases. However, it is essential to analyze the long-term impact of these technologies and avoid a one-size-fits-all approach to care delivery [67,68].

AI systems excel in leveraging vast datasets to perform cost–benefit analyses for tailored monitoring and treatment strategies. For example, in anticoagulation management for patients with atrial fibrillation or artificial heart valves, AI models can predict adverse event risks based on patient data and recommend optimized dosing strategies. Such models could improve patient safety by reducing reliance on routine INR monitoring or addressing complex cases where anticoagulant adjustment is challenging [68,69,70].

In cardiac surgery, high-risk patients—such as those with heart failure undergoing emergency procedures—stand to benefit most from AI-driven continuous monitoring and decision support. These systems could assess risks throughout the hospital stay and post-discharge, potentially preventing adverse events and identifying the need for readmission. Although such comprehensive AI systems do not yet exist, future models combining natural language processing of electronic health records with machine learning could provide precise risk predictions and personalized care for heterogeneous patient populations [71,72,73].

AI offers transformative potential in real-time monitoring and decision support for cardiac surgery. By improving the safety and effectiveness of care, especially for high-risk patients, these technologies can significantly enhance outcomes when coupled with ongoing evaluation and adaptation.

#### 3.1.5. Augmented Cognition and Computer Vision in the Operating Room

Advances in technology have transformed the operating room (OR) into a high-tech environment where AI optimizes workflows and enhances human cognition at individual and team levels. AI systems integrate real-time data to support complex socio-technical tasks in cardiothoracic surgery, aiding cognitive functions such as decision making, situational awareness, and coordination. By adapting to real-time contextual information and predicting future states, AI enhances team performance during intricate procedures [30,32].

Monitoring cognitive states using physiological metrics such as heart rate variability (HRV), electroencephalography (EEG), and near-infrared spectroscopy (NIRS) allows AI systems to provide context-aware assistance. These tools are crucial for managing the dynamic and high-stakes environment of the OR, ensuring optimal team collaboration and reducing cognitive overload [30,32].

Computer vision (CV), a branch of AI that interprets visual data from images and videos, further enhances surgical precision and teamwork. CV applications include workflow segmentation, instrument recognition, and image-guided interventions. In cardiothoracic surgery, CV tracks body positions and movements to objectively measure technical skills, team dynamics, and coordination. By analyzing these metrics, CV contributes to the emerging field of “cognitive surgery”, where AI-driven insights enhance both technical and cognitive aspects of surgical performance [33,74,75].

By integrating augmented cognition and computer vision, AI creates a cohesive framework for optimizing performance in the OR. These technologies not only enhance individual surgical skills but also foster seamless team collaboration, transforming the practice of complex surgeries such as those in cardiothoracic care.

### 3.2. Implementation of Artificial Intelligence in Cardiac Surgery

AI’s integration into cardiac surgery is advancing, with robotics offering some of the most promising applications. Robotic-assisted systems, such as the Da Vinci Surgical System, are currently limited to human-operated procedures, but their potential lies in developing intelligent, autonomous robots capable of adapting to real-time surgical demands [76,77]. Machine learning (ML) models, utilizing both supervised and unsupervised approaches, create predictive systems by analyzing vast datasets, identifying patterns, and making independent decisions with minimal human intervention [78,79].

In cardiac surgery, data from imaging, videos, and physiological metrics are analyzed using AI to enhance diagnoses, prognoses, and treatment plans. While traditional statistical models assess the probabilities of specific outcomes, AI leverages pattern recognition and deep learning to offer more precise and automated insights, particularly for complex cases [79,80,81].

#### 3.2.1. Preoperative Diagnostic Assistance

AI is transforming preoperative diagnostics in cardiothoracic surgery, particularly through imaging-based applications. Machine learning models have demonstrated high accuracy in identifying thoracic pathologies and valvular heart diseases (VHDs) [33,82]. Advanced tools such as AI-enabled cardiac auscultation enhance VHD diagnosis by analyzing heart sounds with techniques such as segmentation, feature extraction, and classification. Digital stethoscopes and deep learning models, such ashidden Markov models (HMMs), have significantly improved the precision and reliability of auscultation, addressing limitations faced by less experienced clinicians [83,84,85].

AI-assisted electrocardiography (AI-ECG) has also shown promise as a large-scale screening tool for early VHD detection. Recent studies on deep learning models (DLMs) identified various VHDs, achieving AUCs of 0.77–0.84 and highlighting their potential for screening and echocardiographic follow-ups [86].

Echocardiography, a cornerstone in cardiac diagnostics, has benefited from AI tools that automate tasks such as image acquisition, view classification, and disease identification. AI-guided echocardiography improves image quality, even in non-expert settings, while convolutional neural networks (CNNs) assist in identifying regional wall motion abnormalities and measuring left ventricular function. These advancements enhance precision, reproducibility, and diagnostic accuracy for conditions such as VHD and aortic diameter abnormalities [83,87,88,89]. AI integration into 3D echocardiographic analysis has shown high reproducibility and reduced variability in assessments of mitral valve geometry, supporting clinical decision making in minimally invasive interventions. Tools such aseSie Valve Software streamline workflows and reduce manual workloads, with ongoing developments aimed at refining automation and improving adaptability [90].

AI is also revolutionizing cardiac CT, especially for procedures such as transcatheter aortic valve implantation (TAVI). Automated algorithms enable precise assessments of valve calcification, annulus geometry, and prosthesis selection, reducing procedural time and complications. Patient-specific 3D modeling further enhances preoperative planning, leading to better outcomes [34,87,91,92].

Similarly, cardiac magnetic resonance (CMR) is evolving with AI-based segmentation tools that automate the quantification of cardiac chamber volumes and regurgitant jets. AI advancements in CMR include automated cardiac chamber segmentation, with large datasets and commercial software such as SuiteHEART^®^ supporting automated biventricular volume and function measurements. Although still developing, deep learning models, such as those for identifying bicuspid aortic valves, show promise in enhancing diagnostic precision for complex VHD cases [93,94,95].

AI-driven preoperative diagnostic tools are streamlining processes, improving accuracy, and enabling personalized care in cardiothoracic surgery. These advancements, from echocardiography to cardiac CT and CMR, are shaping a future where technology plays an integral role in clinical decision making.

#### 3.2.2. Data Collection and Analysis

Systematic and robust data collection has the potential to revolutionize cardiac surgery by enabling objective analysis of surgical outcomes and treatment comparisons. This approach can enhance quality control, evaluate cost-effectiveness, and provide the foundation for AI-driven decision-making tools. AI could facilitate randomized controlled trial simulations with virtual patients, offering a safe and cost-effective method for testing new treatments before clinical implementation [96,97,98,99].

Surgical Data Science (SDS) has emerged as a new discipline focused on enhancing the quality and value of interventional healthcare. By capturing, organizing, and modeling data from sources such as patients, providers, and sensors, SDS leverages AI and machine learning (ML) to identify complex relationships between variables with minimal human input. Unlike traditional regression models, SDS employs advanced unsupervised learning techniques to uncover patterns and improve surgical decision making and patient safety [30,66,100,101].

Big Data analytics, combined with AI and ML, is transforming modern medicine by extracting hidden patterns from electronic health records, imaging, registries, and OMICS data. In cardiac surgery, these tools enable the development of predictive models that provide individualized risk assessments and prognoses, surpassing traditional risk scores. However, while these advancements fuel personalized medicine, randomized controlled trials (RCTs) and meta-analyses remain the cornerstone of clinical guidelines, and the full impact of Big Data on cardiac surgery is still evolving [102].

AI offers a pathway to transition from retrospective, biased data collection to prospective, standardized systems that are comprehensive across all patient populations. Such systems can feed into AI-powered decision support tools that predict outcomes for specific interventions and quantify expected benefits for individual patients. These tools could transform shared decision making between patients and clinicians, providing critical insights during times of economic austerity. Additionally, a national registry incorporating these data systems could serve as a valuable resource for research and healthcare planning, particularly in the UK’s National Health Service [31,103,104].

AI-driven data collection and analysis have the potential to significantly enhance the precision and quality of care in cardiac surgery. By enabling predictive analytics, improving risk stratification, and supporting shared decision making, these technologies pave the way for a more personalized and efficient healthcare system.

#### 3.2.3. Machine Learning Algorithms

Machine learning (ML) algorithms, a key component of artificial intelligence, enable systems to learn from data, identify patterns, and make decisions with minimal human input. Recent advancements have brought ML to the forefront of AI research, particularly in simulating cardiovascular physiology to better understand diseases and treatments. The ultimate goal is a virtual physiological human—a comprehensive digital model of the human body for simulating diseases and aiding clinical decision making [105,106].

Advanced ML approaches, such as inductive learning, analyze large datasets to uncover insights, described by AI pioneer David Spiegelhalter as finding “needles of benefit in immense haystacks of data”. These models complement clinical judgment by predicting disease risks and outcomes based on patient-specific variables, ensuring consistent, evidence-based care and reducing human error [107,108].

Simpler rule-based systems, such as the Calgary REMATCH rule for heart failure readmissions, follow IF-THEN logic but lack flexibility for complex patients with multiple comorbidities. More sophisticated ML techniques, such as supervised and unsupervised learning, overcome these limitations by analyzing diverse datasets to generate tailored insights, advancing personalized care in cardiology [109,110].

#### 3.2.4. Robotic-Assisted Surgery

Robotic technology is transforming surgery, particularly in cardiothoracic procedures, with advancements in autonomous and semi-autonomous systems enabling complex maneuvers such as shared-control in beating-heart surgery. Interdisciplinary innovations, including nanorobots and machine-learning (ML)-empowered instruments, are driving these developments. ML techniques such as imitation learning, hidden Markov models, and neural networks model human surgical skills from recorded data, advancing robotic-assisted surgery’s capabilities [30,111,112].

Robotic-assisted surgery, an advanced form of minimally invasive surgery, is well established in cardiothoracic care. Systems such as the Da Vinci Surgical System allow surgeons to perform tasks in real-time via robotic arms guided by highly detailed 3D imaging. The minimally invasive nature reduces trauma, shortens recovery times, and improves postoperative outcomes compared with open-heart surgery. Enhanced 3D imaging provides better visualization of anatomical structures, particularly in complex areas such as the heart’s conducting system, and aids in preoperative planning. Future integration with virtual reality could further enhance precision and planning [113,114].

As robotic technologies advance, partial or fully autonomous surgeries may reduce costs and workloads, though these benefits must be balanced against potential drawbacks, such as reduced training opportunities for surgeons. Quality improvement tools such as the OR black box system, which captures intraoperative data for analysis, demonstrate how AI can study technical and non-technical surgical performance to improve outcomes. Recent studies also highlight the feasibility of ML algorithms in predicting complications such as hypotension and hypoxemia during cardiothoracic surgeries [30,115].

In summary, robotic-assisted surgery combines precision, efficiency, and reduced patient trauma, with ongoing advancements poised to further revolutionize surgical practice.

#### 3.2.5. Postoperative Management

AI is transforming postoperative management in cardiac surgery by enabling personalized risk assessments and enhancing clinical decision making through machine learning (ML). Supervised ML models provide accurate, predictive, and reproducible evaluations of postoperative risks, improving patient outcomes and healthcare efficiency [33].

For example, Chang et al. employed a Naïve Bayes (NB)-assisted prediction system to assess needs for high-concentration oxygen, ICU care, and ventilator support after lung resection surgery. Patients reported improved understanding of their risks, showcasing the value of digitalized predictions [116]. Similarly, Tseng et al. developed ML models using 94 pre- and intraoperative features to predict cardiac surgery-associated acute kidney injury (CSA-AKI), improving accuracy by preserving variability in continuous data and highlighting the importance of intraoperative time-series data [35].

Incorporating cardiopulmonary bypass-specific intraoperative hypotension (CBP-specific IOH) into preoperative risk models, Fernandes et al. demonstrated improved predictive accuracy for post-surgical mortality. This highlights the value of integrating intraoperative parameters into risk assessments [36]. Additionally, Mufti et al. applied ML to predict postoperative delirium, uncovering hidden patterns that improved prediction accuracy [37].

AI also facilitates remote monitoring of patients with conditions such as coronary artery disease and atrial fibrillation, reducing healthcare costs and advancing precision medicine. By enabling real-time tracking and tailored interventions, AI tools offer significant potential to enhance postoperative care and outcomes [117].

AI’s integration into cardiac surgery not only improves clinical precision but also significantly enhances patient-reported outcomes, such as satisfaction and quality of life. For instance, robotic-assisted cardiac surgery has demonstrated tangible benefits for patients, including reduced postoperative pain, shorter hospital stays, and quicker recovery times, all of which contribute to higher levels of satisfaction [111,113,114,115]. AI-enabled remote monitoring systems further empower patients by offering real-time insights into their recovery, fostering better adherence to rehabilitation protocols, and improving their confidence in managing their health. Moreover, AI’s predictive capabilities, such as identifying early signs of complications, allow for timely interventions that mitigate anxiety and enhance long-term quality of life [117]. These advancements underscore AI’s transformative potential not only in improving surgical outcomes but also in addressing the broader needs and preferences of patients, ultimately contributing to more holistic and patient-centered care.

**Figure 2:**
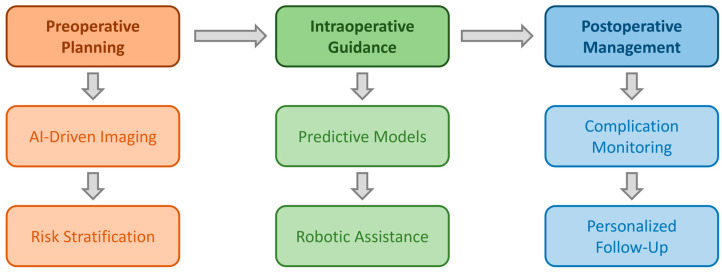
This diagram illustrates the integration of AI across the cardiac surgery workflow, encompassing preoperative planning, intraoperative guidance, and postoperative management. Key applications include AI-driven imaging and risk stratification during preoperative planning, predictive models and robotic assistance for intraoperative guidance, and real-time complication monitoring and personalized follow-up care in postoperative management. The arrows represent the sequential flow of care phases, highlighting the comprehensive role of AI in enhancing precision, decision making, and patient outcomes throughout the continuum of cardiac surgery.

Figure 2 provides a visual representation of the multifaceted benefits of integrating artificial intelligence (AI) into cardiac surgery, highlighting its transformative role across preoperative planning, intraoperative guidance, and postoperative management.

### 3.3. Risks and Challenges of Artificial Intelligence in Cardiac Surgery

While artificial intelligence (AI) holds transformative potential in cardiac surgery, its implementation must be approached cautiously due to validation, ethical, and medicolegal challenges. AI should complement, not replace, human expertise, with essential oversight to mitigate risks. Cardiac surgery has been slow to adopt new technologies, reflecting the high stakes and skepticism surrounding critical cardiac disease management [38].

The closer AI applications are to direct patient outcomes, the higher the associated risks. For example, robotic systems performing surgery or ML-based models predicting postoperative outcomes carry significant risks of adverse events. These events may result from incorrect AI decisions or errors in their interpretation, potentially leading to morbidity or mortality [30,33,39,118,119].

#### 3.3.1. Ethical Considerations

The ethical challenges of AI in cardiac surgery include the need for a moral and legal framework to guide decision making. Differing opinions on appropriate ethical standards complicate this task, particularly as these frameworks may not align with those programmed into AI systems [120,121,122].

Accountability is another critical concern. In current practice, physicians bear responsibility for patient outcomes, but with AI-driven decisions, it is unclear who would be held accountable for mistakes leading to patient harm. Moreover, understanding why an AI made a particular decision can be challenging, especially in complex scenarios. Unlike human errors, which can often be traced to specific causes such as lack of experience or fatigue, AI decisions may lack transparency, undermining trust in critical situations [8,103,120].

Society must also address broader ethical concerns. While AI systems can make decisions faster than humans, speed does not guarantee accuracy. Rapid but incorrect decisions could jeopardize patient safety, highlighting the importance of thorough validation and oversight during implementation [122,123,124].

#### 3.3.2. Data Privacy and Security

The integration of AI into healthcare raises significant concerns about data privacy and security. Many healthcare institutions lack the IT tools and expertise needed to protect clinical data from theft or corruption. While cybersecurity threats such as hacking are a clear risk, subtler breaches of data integrity may be harder to detect and address. The growing use of clinical data for AI training and testing, particularly when shared across institutions or with private companies, increases exposure to regulatory and legal vulnerabilities [121,125].

The use of personal data in AI-driven medical predictions also introduces legal complexities. Data used to train AI models may later become subject to subpoenas in legal cases, requiring its retention and potential disclosure. Additionally, as AI becomes a core component of medical data systems, these systems must ensure the perpetual storage of patient data, even as storage technologies evolve, to maintain continuity in patient care and health research [121,125].

AI-specific policies for data governance and secure storage are essential to address these challenges. Such policies must balance the need for robust data protection with the demands of advancing medical research and clinical care.

#### 3.3.3. Reliability and Trustworthiness of AI Systems

The reliability of AI systems in cardiac surgery depends on their ability to generalize across diverse datasets while avoiding issues such as overfitting or underfitting. Machine learning models analyze data patterns to make predictions, but their accuracy is constrained by the quality and relevance of the data provided. Insufficient data quantity or poor data quality remains a major barrier to successful AI implementation for many clinical problems [126,127,128,129]. Improving the reliability of AI requires better quantification of data, such as using advanced biomarkers or imaging techniques, and ensuring the data align with clinical decision-making contexts. Ultimately, AI decisions are only as good as the data that inform them.

A notable example of successful AI implementation is IBM Watson, which provided treatment recommendations for breast cancer based on patient case notes, history, and research data. In clinical trials, Watson’s recommendations aligned more closely with national guidelines and research papers than treatments selected without AI assistance, demonstrating AI’s potential to improve adherence to evidence-based care [130,131].

In cardiac surgery, AI aims to improve patient outcomes by integrating quantitative data into clinical decision making. For example, AI systems could help determine the best management strategies for valvular heart disease based on patient-specific factors such as severity, age, and comorbidities. These systems could also guide optimal timing for follow-up tests or interventions, helping clinicians balance risks and benefits effectively. However, the superiority of AI-informed decisions over traditional approaches in cardiology and cardiac surgery remains largely unproven, underlining the need for further research and validation [132,133,134].

#### 3.3.4. Illustrating Challenges with AI in Cardiac Surgery

While AI offers transformative potential in cardiac surgery, it is crucial to address real-world challenges and limitations through concrete examples [128,129]. Instances of AI system failures or near-misses underline the importance of robust testing, validation, and ethical oversight. For example, AI systems have been known to misclassify diagnostic imaging due to biased training datasets, potentially leading to incorrect surgical planning or delayed interventions. A notable case involves a machine learning algorithm for postoperative risk prediction that underestimated complication risks in underrepresented patient populations, resulting in suboptimal postoperative monitoring. These examples highlight the importance of diverse and high-quality training datasets, transparency in algorithm development, and human oversight to mitigate risks. Integrating lessons from such cases can strengthen the safe adoption of AI in cardiac surgery, ensuring that the technology complements rather than undermines clinical expertise [135,136,137,138,139].

For case studies, specific examples of AI-related issues in cardiac surgery may not yet be well-documented in the literature, as this is an emerging field. However, analogous cases in other surgical specialties or healthcare areas could provide valuable insights.

##### AI Diagnostic Failures in Radiology

In radiology, AI applications have sometimes led to diagnostic inaccuracies due to algorithmic limitations or insufficient training data. For example, a study highlighted that AI models could misinterpret medical images, resulting in false positives or negatives, which may adversely affect patient care [135,136].

##### Near-Misses in Robotic Surgery

In robotic-assisted surgeries, AI integration has occasionally encountered challenges requiring human intervention. A review discussed how AI-driven robotic systems might face difficulties during complex procedures, emphasizing the necessity for continuous human oversight to manage unexpected intraoperative events [137,138,139].

These examples demonstrate the importance of comprehensive validation, diverse training datasets, and maintaining human oversight when implementing AI in cardiac surgery. Addressing these challenges is crucial to harness AI’s benefits while mitigating potential risks.

### 3.4. Future Directions and Research Opportunities

The integration of artificial intelligence (AI) into cardiac surgery offers vast potential for optimizing patient care, surgical procedures, and healthcare management. Traditionally, statistical methods have dominated data analysis in healthcare, but recent advances in AI open new possibilities, from surgery scheduling and bed management to personalized predictions of surgical outcomes and the development of AI-assisted patient companions. The foundation of these advancements lies in the wealth of data generated from patient records, imaging studies, and clinical trials, which align with the growing emphasis on evidence-based medicine and fully informed patient consent [140,141].

One of the most promising future directions is the development of intelligent decision support systems (DSS). By leveraging AI, these systems can analyze vast datasets to provide actionable insights for clinicians, reducing medical errors and improving outcomes. A recent NHS study highlighted the need for such technologies, reporting that one in ten patients experience a medical error, with half of these being preventable [140]. AI-driven DSS could not only enhance diagnostic accuracy but also refine intraoperative decision making and postoperative management, paving the way for safer, more efficient surgical care.

#### 3.4.1. Advances in AI-Driven Robotics

The increased adoption of minimally invasive surgery has driven the development of robotic-assisted systems, and the future of robotics in cardiac surgery appears increasingly autonomous. Intelligent robotic devices with advanced image recognition and tracking capabilities could perform precise repair or reconstructive procedures independently, reducing risks associated with human error and enhancing surgical precision. These systems could incorporate real-time data analysis to adapt intraoperatively, making them indispensable for complex or high-risk cases [142,143,144].

#### 3.4.2. Predictive and Personalized Care

AI offers immense potential to revolutionize preoperative planning and risk prediction. Future AI tools could integrate genomic, proteomic, and metabolomic data into clinical decision making, ushering in an era of personalized care. For example, machine learning models could predict long-term surgical outcomes, recommend tailored interventions, and guide the timing of non-invasive or invasive testing. Additionally, AI companions could support patients postoperatively, providing personalized rehabilitation plans and monitoring for early signs of complications [40,41,144,145].

#### 3.4.3. AI in Healthcare System Optimization

Beyond direct patient care, AI could transform healthcare systems by optimizing resource allocation. Advanced predictive algorithms may improve scheduling efficiency for surgeries and ICU bed management, reduce waiting times, and streamline patient workflows. AI could also enable predictive maintenance of surgical equipment, reducing downtime and ensuring operational continuity [42].

#### 3.4.4. Emerging Technologies and Interdisciplinary Research

The future of AI in cardiac surgery will likely involve collaborations across disciplines such as bioinformatics, nanotechnology, and computational modeling. Nanorobotics holds particular promise, with potential applications in targeted drug delivery or micro-scale surgical interventions within the cardiovascular system [43,146]. Furthermore, advances in augmented reality (AR) and virtual reality (VR) could enhance surgical training and preoperative planning, providing immersive environments for surgeons to rehearse complex procedures. AI could integrate with AR/VR platforms to offer real-time, context-aware guidance during surgeries [147].

#### 3.4.5. Transforming Roles of Surgeons and Healthcare Professionals

The integration of artificial intelligence (AI) into cardiac surgery is poised to significantly reshape the roles of surgeons and healthcare professionals. As AI systems take on tasks such as risk stratification, surgical planning, and real-time decision support, surgeons may transition from primary operators to supervisors overseeing AI-assisted procedures [2,68,148]. This shift could enable them to focus on complex decision making and managing atypical cases that fall outside AI’s programmed parameters. Similarly, healthcare professionals may see their roles evolve, with an increased emphasis on managing AI-driven systems, interpreting data outputs, and maintaining the human connection in patient care. While these advancements promise to enhance efficiency and precision, they also raise workforce challenges, such as the need for specialized training in AI systems and potential reductions in demand for certain manual tasks [33,149]. Ensuring a smooth transition will require interdisciplinary collaboration, robust training programs, and proactive strategies to address ethical, regulatory, and workforce implications [1,40,150].

#### 3.4.6. Ethical and Regulatory Considerations

To fully realize the potential of AI in cardiac surgery, addressing ethical and regulatory challenges will be critical. Developing robust frameworks for data privacy, security, and algorithm accountability is essential to ensure patient safety and trust. Future research should focus on explainable AI (XAI), enabling clinicians to understand the rationale behind AI-driven decisions, particularly in high-stakes scenarios [120,122].

This extensive review of the literature concludes that currently the use of AI in cardiac surgery has primarily been to predict postoperative complications and to enhance clinicians’ decision making through improved preoperative risk assessment, stratification, and prognostication. Although significant advancements in AI applications within cardiac surgery have been made over the past decade, further research is required to validate its accuracy and ensure its safety for clinical implementation [151]. The future of AI in cardiac surgery is poised for transformative growth, with advancements in robotics, predictive analytics, and personalized care leading the way. While significant progress has been made, the field remains in its infancy, offering countless opportunities for innovation and interdisciplinary collaboration. By addressing ethical, regulatory, and technical challenges, AI has the potential to redefine cardiac surgery, delivering safer, more effective, and patient-centered care [44,151,152].

## 4. Limitations

Despite the transformative potential of artificial intelligence (AI) in cardiac surgery, several limitations must be acknowledged.

Data quality and generalizability: The effectiveness of AI systems depends heavily on the quality and diversity of data used for training. Many existing models are trained on limited datasets, which may not represent broader populations or account for variability in clinical settings. This limits the generalizability of AI tools across different healthcare systems and patient demographics.

Validation and external testing: While promising, many AI applications in cardiac surgery lack sufficient external validation. Models are often optimized on retrospective data, which may not reflect real-world conditions. Prospective, multi-center trials are needed to confirm their reliability and effectiveness in clinical practice.

Integration challenges: The integration of AI into routine surgical workflows remains complex, requiring significant adaptation of existing systems, staff training, and financial investment. Resistance to adopting AI tools among clinicians, due to concerns over trust, usability, and potential workflow disruptions, further complicates implementation.

Ethical and regulatory concerns: AI systems raise ethical questions about accountability, transparency, and fairness. Unclear regulatory frameworks and the lack of guidelines for AI use in healthcare impede widespread adoption. Ensuring compliance with data privacy and security standards adds another layer of complexity.

Algorithm bias: AI models are susceptible to bias introduced by imbalanced or incomplete training datasets. Such biases could lead to inaccurate predictions, particularly for underrepresented patient groups, exacerbating healthcare disparities.

Limited scope of current applications: Current AI tools are highly specialized, focusing on isolated tasks such as risk stratification, imaging analysis, or decision support. Achieving comprehensive, integrated AI systems that can address the full spectrum of perioperative care remains a distant goal.

Resource limitations: Developing and deploying AI systems requires significant computational resources, infrastructure, and expertise, which may not be available in resource-limited healthcare settings. This disparity could hinder equitable access to AI-driven solutions globally.

Unintended consequences: Overreliance on AI could lead to unintended consequences, such as the erosion of clinical expertise or overconfidence in algorithmic recommendations. Continuous monitoring and oversight are essential to mitigate these risks.

Addressing these limitations will require ongoing research, collaboration, and iterative refinement of AI systems to ensure their safe, equitable, and effective integration into cardiac surgery and broader healthcare settings.

## 5. Conclusions

This review highlights the transformative potential of artificial intelligence (AI) in cardiac surgery, showcasing its applications in risk stratification, surgical planning, real-time decision support, and postoperative management. AI-driven tools such as machine learning algorithms, robotic-assisted systems, and predictive models are advancing precision, efficiency, and patient outcomes in this high-risk field. However, significant challenges, including data quality, validation, integration, and ethical considerations, must be addressed to ensure safe and effective implementation. The findings emphasize the need for robust interdisciplinary collaboration, rigorous testing, and targeted innovation to maximize AI’s benefits in cardiac surgery. By bridging technology with clinical expertise, AI holds the potential to revolutionize cardiac surgery, paving the way for safer, more personalized, and efficient care.

## Figures and Tables

**Table 1:** Summary of key studies on artificial intelligence applications in cardiac surgery. The table includes reference numbers, authors, publication years, key outcomes, and their relevance to this review, highlighting advancements in risk prediction, surgical planning, intraoperative guidance, and postoperative management.

Reference Number	Authors	Year	Key Outcomes and Contributions	Relevance to Review
[9]	Allyn J, et al.	2017	Compared ML models with EuroSCORE II for mortality prediction after elective cardiac surgery. ML showed superior predictive capabilities.	Directly evaluates AI’s clinical utility in cardiac surgery outcomes.
[10]	Nashef SAM, Ali J	2023	Explored AI-based risk assessment models for cardiac surgery, emphasizing accuracy and clinical applicability.	Relevant to risk modeling and decision making in cardiac surgery.
[11]	Poullis M	2024	Discussed AI’s transformation of risk modeling in cardiac and thoracic surgery, highlighting predictive advances.	Key for understanding AI’s role in predictive risk assessment in this field.
[12]	Lee HC, et al.	2018	Developed and validated ML models for predicting acute kidney injury after cardiac surgery with high accuracy.	Provides critical insights into AI’s impact on postoperative management.
[13]	Palmieri V, Montisci A, Vietri MT, et al.	2023	AI applications in heart transplantation, with emphasis on predictive modeling for patient outcomes and diagnostic tools.	Provides insights into the integration of AI in specialized cardiac surgery contexts.
[14]	Agasthi P, Buras MR, Smith SD, et al.	2020	Explored ML tools for long-term graft survival prediction in heart transplantation.	Relevant to extending AI applications to long-term postoperative outcomes in cardiac surgery.
[15]	Penny-Dimri JC, et al.	2022	Systematic review of ML methods for predicting adverse outcomes in cardiac surgery, showing promise but limited by data and study design.	Offers a meta-perspective on AI applications in predicting surgical complications.
[16]	Bodenhofer U, et al.	2021	Demonstrated that ML-based tools outperform EuroSCORE for risk classification in heart valve surgery.	Directly relevant to improving preoperative risk stratification with AI.
[17]	Shuhaiber JH, Conte JV	2021	Examined ML applications in heart valve surgery, focusing on predictive analytics for outcomes and complications.	Directly applicable to improving precision in valve-related surgical interventions.
[18]	Gomes B, et al.	2021	Developed ML-based risk prediction for intrahospital outcomes in TAVI, showing superior accuracy over traditional scores.	Highlights AI’s role in precision risk assessment for TAVI.
[19]	Kalisnik JM, et al.	2022	Developed an AI model for early detection of acute kidney injury post-cardiac surgery, achieving high accuracy.	Critical for postoperative monitoring and complication prevention with AI.
[20]	Fliegenschmidt J, et al.	2021	Demonstrated AI’s potential for predicting postoperative delirium in cardiac surgery patients.	Expands AI’s applications to cognitive outcome monitoring post-surgery.
[21]	Naruka V, et al.	2022	Systematic review of ML and AI in cardiac transplantation, emphasizing predictive modeling for graft survival.	Highlights AI’s potential in complex and specialized cardiac interventions.
[22]	Lisboa PJG, et al.	2022	Developed explainable AI models for predicting survival in heart transplantation.	Relevant for advancing transparency and accuracy in AI predictions in cardiac surgery.
[23]	Lin Y, et al.	2022	Used AI-based logistic regression for predicting rupture risk in acute aortic dissection patients.	Highlights AI’s role in high-stakes preoperative decision making.
[24]	Ostberg NP, et al.	2023	Applied ML to predict complications in thoracoabdominal aortic aneurysms.	Expands AI applications to complex vascular conditions managed in cardiac surgery.
[25]	Guo T, et al.	2021	Developed ML models for predicting in-hospital mortality in acute aortic dissection.	Highlights AI’s potential in improving acute care decision making in cardiac surgery.
[26]	Farooqi HA, Nabi R	2024	Discussed novel AI and ML approaches to reducing mortality in acute type A aortic dissection.	Highlights innovative approaches in critical cardiac conditions.
[27]	Varpaei HA, Robbins LB, Ling J, et al.	2024	Scoping review on AI-related cognitive dysfunction after cardiothoracic surgery.	Examines postoperative cognitive outcomes, a less explored AI application in cardiac surgery.
[28]	Li G, Wang H, Zhang M, et al.	2021	Predicted 3D cardiovascular hemodynamics before and after coronary artery bypass using deep learning techniques.	Demonstrates AI’s role in personalized surgical planning and postoperative assessments.
[29]	Ferrari E, Gallo M, Wang C, et al.	2020	Explored AI and 3D printing for procedural planning, teaching, and innovation in cardiovascular surgeries.	Highlights AI’s role in advanced visualization and surgical preparation.
[30]	Dias RD, Shah JA, Zenati MA	2020	Discussed AI’s role in cardiothoracic surgery, focusing on real-time decision support and workflow optimization.	Broadly relevant to all facets of AI integration in cardiac surgery.
[31]	Jones B, et al.	2020	Reviewed autonomously driven AI applications in cardiothoracic surgery, focusing on real-time guidance and precision.	Expands understanding of AI’s autonomy in surgical practices.
[32]	Zenati MA, et al.	2020	Examined cognitive engineering and AI to enhance patient safety and outcomes in cardiothoracic surgery.	Explores interdisciplinary approaches combining AI and cognitive science in surgery.
[33]	Mumtaz H, et al.	2022	Discussed future directions of AI in cardiothoracic surgery, with focus on surgical innovation and patient outcomes.	Provides forward-looking perspectives on AI applications.
[34]	Lalys F, et al.	2019	Developed automatic aortic root segmentation and anatomical landmark detection for TAVI procedural planning using AI.	Demonstrates AI’s utility in precision planning for complex cardiac interventions.
[35]	Tseng PY, et al.	2020	Developed ML models using intraoperative and preoperative data to predict cardiac surgery-associated acute kidney injury.	Highlights AI’s role in enhancing postoperative complication management.
[36]	Fernandes MPB, et al.	2021	Integrated preoperative and intraoperative data into ML models to predict mortality after cardiac surgery.	Showcases the impact of AI in improving surgical mortality predictions.
[37]	Mufti HN, et al.	2019	Exploited ML algorithms to predict postoperative delirium following cardiac surgery.	Relevant to improving postoperative cognitive outcome predictions.
[38]	Clark SC	2024	Explored the transformative role of AI tools such as ChatGPT in cardiac surgery and heart transplantation.	Highlights the potential integration of conversational AI in surgery and training.
[39]	Nedadur R, et al.	2024	Examined emerging AI tools in cardiac surgery, with a focus on clinical applications and future potential.	Discusses cutting-edge advancements in AI for cardiac surgery.
[40]	Rad AA, et al.	2022	Reviewed applications of virtual and augmented reality in cardiac surgery.	Expands on future technologies integrating with AI in cardiac surgery.
[41]	Sulague RM, et al.	2024	Systematic review of AI applications in cardiac surgery, focusing on risk prediction, surgical accuracy, and clinical outcomes.	Comprehensive summary of AI’s utility across various phases of cardiac surgery.
[42]	Bhushan R, Grover V	2024	Reviewed the current understanding of AI’s impact on cardiac surgery, with an emphasis on predictive and robotic technologies.	Provides a systematic overview of AI advancements relevant to cardiac surgery.
[43]	Mestres CA, et al.	2022	Highlighted AI’s potential for predicting surgical outcomes, emphasizing the need for validation and clinical integration.	Discusses challenges and opportunities for AI adoption in outcome prediction.

## Data Availability

The data presented in this study are available upon request from the corresponding author.
